# O-GlcNAcylation Negatively Regulates Cardiomyogenic Fate in Adult Mouse Cardiac Mesenchymal Stromal Cells

**DOI:** 10.1371/journal.pone.0142939

**Published:** 2015-11-13

**Authors:** Ayesha Zafir, James A. Bradley, Bethany W. Long, Senthilkumar Muthusamy, Qianhong Li, Bradford G. Hill, Marcin Wysoczynski, Sumanth D. Prabhu, Aruni Bhatnagar, Roberto Bolli, Steven P. Jones

**Affiliations:** Institute of Molecular Cardiology; Diabetes and Obesity Center, Department of Medicine, Division of Cardiovascular Medicine, University of Louisville, Louisville, Kentucky, United States of America; Georgia Regents University, UNITED STATES

## Abstract

In both preclinical and clinical studies, cell transplantation of several cell types is used to promote repair of damaged organs and tissues. Nevertheless, despite the widespread use of such strategies, there remains little understanding of how the efficacy of cell therapy is regulated. We showed previously that augmentation of a unique, metabolically derived stress signal (i.e., O-GlcNAc) improves survival of cardiac mesenchymal stromal cells; however, it is not known whether enhancing O-GlcNAcylation affects lineage commitment or other aspects of cell competency. In this study, we assessed the role of O-GlcNAc in differentiation of cardiac mesenchymal stromal cells. Exposure of these cells to routine differentiation protocols in culture increased markers of the cardiomyogenic lineage such as Nkx2.5 and connexin 40, and augmented the abundance of transcripts associated with endothelial and fibroblast cell fates. Differentiation significantly decreased the abundance of O-GlcNAcylated proteins. To determine if O-GlcNAc is involved in stromal cell differentiation, O-GlcNAcylation was increased pharmacologically during the differentiation protocol. Although elevated O-GlcNAc levels did not significantly affect fibroblast and endothelial marker expression, acquisition of cardiomyocyte markers was limited. In addition, increasing O-GlcNAcylation further elevated smooth muscle actin expression. In addition to lineage commitment, we also evaluated proliferation and migration, and found that increasing O-GlcNAcylation did not significantly affect either; however, we found that O-GlcNAc transferase—the protein responsible for adding O-GlcNAc to proteins—is at least partially required for maintaining cellular proliferative and migratory capacities. We conclude that O-GlcNAcylation contributes significantly to cardiac mesenchymal stromal cell lineage and function. O-GlcNAcylation and pathological conditions that may affect O-GlcNAc levels (such as diabetes) should be considered carefully in the context of cardiac cell therapy.

## Introduction

Physiological adaptation of cells to environmental cues requires the integration of metabolic signals. Metabolism is linked to physiological functions such as proliferation and differentiation. Mesenchymal/stem cells, in particular, have unique metabolic demands to support their multifarious functions, ranging from dormancy to periods of proliferation or differentiation. Thus, leveraging our understanding of mesenchymal cell metabolism and metabolic signaling may bolster the efficacy of cell therapy.

In addition to energy conversion, metabolism also contributes to metabolic signaling, which in some cases involves posttranslational, glycosyl modifications derived from carbon sources such as glucose and glutamine. In essentially all multi-cellular eukaryotes, a distinct form of O-linked glycosylation—the β-O-linkage of *N*-acetylglucosamine (i.e., O-GlcNAc)[1] to intracellular proteins—regulates numerous cellular functions. Recent studies suggest that protein O-GlcNAcylation could modulate stem cell biology. Indeed, this inducible stress response exerts an essential prosurvival role in adult murine Sca-1^**+**^/lin^**-**^ cardiac mesenchymal stromal cells[2]. Thus, O-GlcNAc-‘priming’ may promote cell retention during adoptive transfer; however, whether protein O-GlcNAcylation influences other factors that could regulate cell function, such as differentiation, is unknown.

Although we have shown that enhanced O-GlcNAcylation is cytoprotective for cardiac mesenchymal cells[2] and cardiac myocytes[3–9], it is not known whether such protection occurs at the expense of differentiation or proliferation. Prior to performing *in vivo* studies with hyper-O-GlcNAcylated mesenchymal cells, it is important to understand how key functional aspects (beyond cell survival) may be affected. In the present study, we subjected adult, murine Sca-1^**+**^/lin^**-**^ cardiac mesenchymal cells to differentiation stimuli to address this question.

## Materials and Methods

### Cell culture and flow cytometric analysis

The University of Louisville Institutional Animal Care and Use Committee reviewed and approved all animal procedures, which were performed in accordance with federal guidelines. Mice were anesthetized with pentobarbital sodium; the hearts were removed for cell isolation, and the animals euthanized by consequent exsanguination under pentobarbital anesthesia. Cells isolated from adult male wild-type (C57BL6, eGFP) or OGT floxed mouse heart outgrowth cultures were subjected to sequential sorting for c-kit^+^/lin^-^ markers using magnetic immunobeads^[^ ^10^ ^]^ and analyzed by flow cytometry. Adult cardiac cells and cellular controls stained with anti-mouse CD105 (APC, Clone MJ7/18; eBioScience), CD90.2 (PE, Clone 30-H12; eBioScience), CD73 (PE, Clone eBioTY/11.8; eBioScience), CD29 (PE, Clone eBioHMb1-1; eBioscience), CD31 (PE, Clone 390; eBioscience), CD45 (PE, Clone 30-F11;BD Pharmingen), CD34 (PE, Clone RAM34; BD Pharmingen), CD117 (APC-eFluor 780, Clone 2B8; eBioscience), Sca-1 (PerCP-Cy5.5, Clone D7; eBioscience antibodies. Data were acquired on a LSRII flow cytometer (BD BioSciences) and analyzed with FlowJo software (v10.0.07). Discrimination gates were set using unstained samples. Adult cardiac mesenchymal cells were cultured in DMEM/F12 containing leukemia inhibitory factor (1000 U/mL), basic fibroblast growth factor (20 ng/mL), epidermal growth factor (20 ng/mL), and 10% embryonic stem cell grade fetal bovine serum, as described^[^ ^2^ ^,^ ^11^ ^]^.

### Pharmacological augmentation of O-GlcNAcylation

To augment O-GlcNAcylation of cellular proteins, cells were treated for 16–18 h with thiamet-G (TMG; 0.025 mmol/L; Cayman Chemical) ^[^ ^12^ ^]^, a potent inhibitor of O-GlcNAcase.

### OGT gene deletion

Replication-deficient adenovirus (Vector Biolabs) carrying the *Cre recombinase* gene (AdCreGFP or AdCMViCre) was transduced into cells carrying loxP-flanked copies of the *Ogt* gene at 500 MOI for 72 h to delete the *Ogt* gene[2]. AdNull was used as a control adenovirus. Functional expression was determined by immunoblot analysis.

### Protein expression

Whole cell lysates were prepared using standard protocols for total cellular protein. 10–25 μg (as appropriate) of protein was resolved by SDS-PAGE to immunoblot on PVDF membranes for detecting protein O-GlcNAcylation^[^ ^2^ ^,^ ^5^ ^]^ (1:1,000; CTD 110.6, Covance) α-smooth muscle actin (1:10,000; clone 1A4, Sigma-Aldrich, Inc.), LC3B II (1:1,000; Cell Signaling Technology) and p62/SQSTM1 (1:500; D5E2, Cell Signaling Technology), followed by the appropriate secondary antibody (Santa Cruz Biotechnology, Inc.). Densitometry was performed on a Fuji LAS-3000 bio-imaging analyzer.

### Differentiation Assay

Cardiac mesenchymal cells were withdrawn from complete culture medium and differentiated in Ham’s F12 (containing 10% fetal bovine serum) with 10^−9^ M dexamethasone[13] for 5 d, at the end of which protein or RNA was harvested for immunoblotting or quantitative RT-PCR, as required.

After withdrawal from complete culture medium, cardiac mesenchymal cells were differentiated in Ham’sF12:IMDM (Iscove's Modified Dulbecco's Medium) (containing 2% horse serum) and exposed to 5 μM 5-Azacytidine for 3 d[14]. Following this, differentiation medium was refreshed at day 4, and from day 6 onward cells were subjected to an alternating treatment with TGFβ (10 ng/mL) and ascorbic acid (10^−4^ M) up to a period of 21 d. Protein or RNA was harvested for immunoblotting or quantitative RT-PCR, as required on days 1, 3, 5, 7 or 21.

### Quantitative Real-time PCR

Total RNA extraction was performed using TRIzol® reagent, following which cDNA was synthesized using the High Capacity cDNA Synthesis Kit according to the manufacturer's instructions (Thermo Fisher Scientific, Inc). Specific primers (indicated in Table 1) were used to detect and analyze mRNA levels with FastSYBR Green (Applied Biosystems); the relative mRNA expression was calculated using the comparative CT (ΔΔ CT) method after normalizing to levels of 18S mRNA.

**Table 1:** Mouse gene-specific primer sequences for quantitative real-time PCR.

Name	Primer	Sequence
Nkx2.5	Forward	5’-CTCCGCCAACAGCAACTTC-3’
Nkx2.5	Reverse	5’-GGACTCTGCACGGTGTTCAA-3’
Connexin 40	Forward	5’-CACAGTCATCGGCAAGGTCT-3’
Connexin 40	Reverse	5’-CTGAATGGTATCGCACCGGA-3’
cTNI	Forward	5’-CCCACCCTCCGAAGAGTGA-3’
cTNI	Reverse	5’-CCAGCAGCGCCTGCAT-3’
VE Cadherin	Forward	5’-CAAGATCAGCTCCTCCACGA-3’
VE Cadherin	Reverse	5’-GTAGCATGTTGGGGGTGTCT-3’
Cardiac Actin	Forward	5’-AGACCACCGCTTTGGTGTGT-3’
Cardiac Actin	Reverse	5’-GCAAAGCCGGCCTTCAC-3’
Col1α	Forward	5’-CCCTGGTCCTCGAGGTCGCA-3’
Col1α	Reverse	5’-TTCTTGCGGCTGCCTTCGGG-3’
Thy1	Forward	5’-CCCTCTGTGCCAGCCCCTCT-3’
Thy1	Reverse	5’-TGGGACAGGCAGAGCTGCCA-3’
18S	Forward	5’-CGAACGTCTGCCCTATCAACTT-3’
18S	Reverse	5’-ACCCGTGGTCACCATGGT-3’

### *Nkx2*.*5* promoter reporter assay

To characterize the effect of elevated O-GlcNAcylation (using TMG) on *Nkx2*.*5* promoter activation, the upstream region of the mouse *Nkx2*.*5* gene (about 1410bp upstream; -1200bp/+210bp) was cloned and transfected into HEK cells treated with Vehicle (Veh) or TMG. In brief, the upstream region of mouse *Nkx2*.*5* gene was PCR-amplified with specific primers containing *Kpn1* and *HindIII* restriction enzyme sites (Sense 5’-aaaGGTACCCCAACTCGTCCTTCATATCTGTGT-3’ and Antisense 5’-aaaAAGCTTGGTGGCGACGCAGGTTTC-3’) using mouse genomic DNA and then cloned into a pGL3-basic luciferase reporter vector (Promega). Small-scale plasmid extraction was performed using the PureYield^TM^ plasmid miniprep system (Promega) and utilized to verify the sequence. For downstream transfection experiments, large-scale plasmid purification was performed using the QIAfilter^TM^ plasmid midi kit (Qiagen). The *Nkx2*.*5* promoter or the promoter-less pGL3 control (500 ng/well of a 12-well plate) were transiently transfected in HEK293 cells using Lipofectamine 2000 (Thermo Fisher Scientific, Inc), while the pRL-CMV *renilla* luciferase control vector (Promega) served as the transfection control (20 ng/well of a 12-well plate). 20 h post-transfection the Dual Luciferase Reporter Assay System (Promega) was used according to the manufacturer’s guidelines to measure *Firefly* and *renilla* luciferase reporter activities via luminescence on a Glomax luminometer (Promega). The measurements from *firefly* luciferase were normalized to *renilla* luciferase and relative light units (RLUs) were calculated after normalizing to the pGL3 control (promoter-less) vector in the Veh group.

### Autophagy flux assessment

Bafilomycin A1 (Sigma-Aldrich, Inc.) was used at a low dose (10^−9^ M) to block the autophagosome-lysosome fusion step[15, 16] 2 h prior to differentiation in Ham’s F12:IMDM (containing 2% horse serum) on day 1 (see above for Differentiation Assay). Accumulation of LC3B was assessed by immunoblotting.

### Proliferation assay

Adult cardiac mesenchymal cells were cultured in complete medium and sequentially harvested at 24, 48, 72 and 96 h for automated counting using a cytometer (BD Accuri™ C6) or manual counts with a hemocytometer. Growth profiles were constructed by plotting the fold increases of cell numbers over time post-modulation of O-GlcNAc levels (see above for pharmacological augmentation of O-GlcNAcylation, OGT gene deletion).

### Transwell migration assay

Adult cardiac mesenchymal cells were harvested post-modulation of O-GlcNAc levels (see above for pharmacological augmentation of O-GlcNAcylation and OGT gene deletion) and introduced in 12-well format cell culture inserts (BD Biosciences) with an 8 μm pore diameter for a Boyden-chamber type migration assay[17, 18]. Cells were allowed to migrate for 16–18 h toward 10% FBS in the outer chamber. The inserts were removed, inner surfaces swabbed to remove unmigrated cells, and processed for staining with Giemsa or crystal violet. Inserts were affixed to glass slides with the lower surface facing upward, cover-slipped and five random images were obtained at 10x by light microscopy. Quantification of migrated cells was performed with ImageJ (version 1.47 8).

### Statistical analyses

Data are reported as means of individual data points, and were analyzed by unpaired t test or ANOVA with post hoc analysis (Bonferroni’s Multiple Comparison Test), as appropriate. Graphs were generated and statistical analyses performed using GraphPad Prism 5.0f. Differences were accepted as significant when p<0.05.

## Results

### Flow cytometric analysis of Sca-1^+^/lin^-^ murine cardiac cells

**Fig 1:**
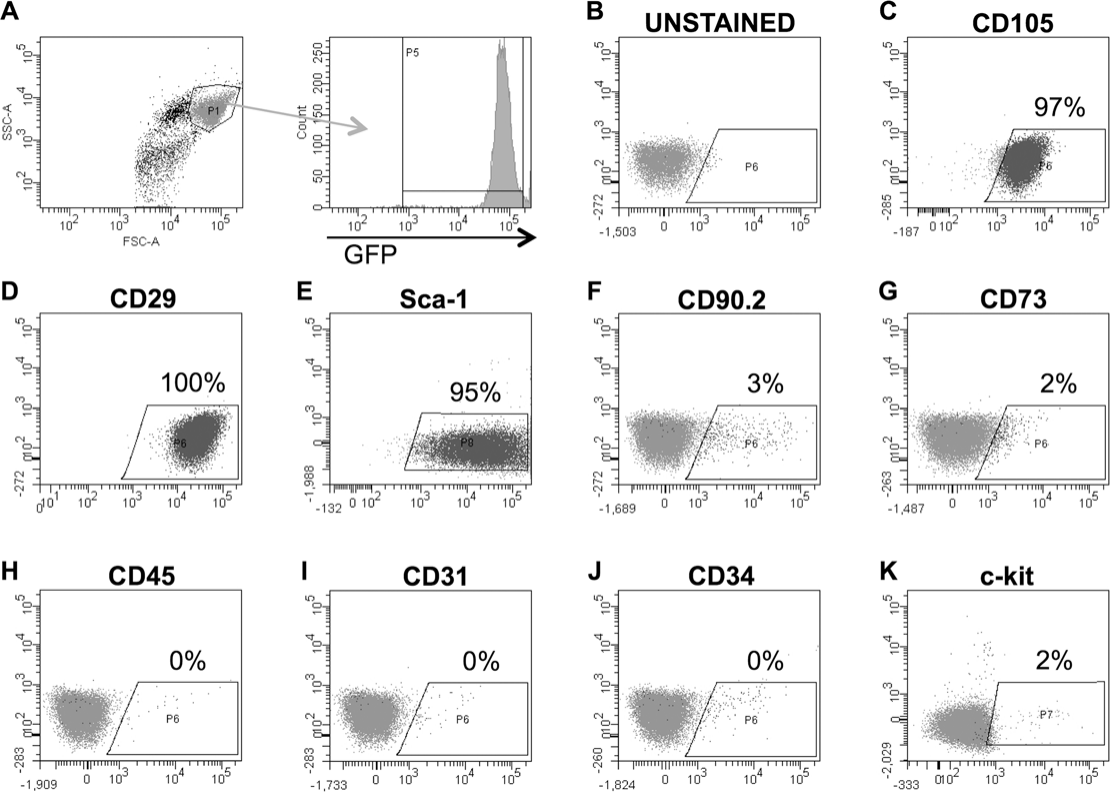
Flow cytometric characterization of Sca-1^+^/lin^-^ murine cardiac cells. **A, B:** Gating strategy, and **C-K:** flow cytometric analyses for **C:** CD105, **D:** CD29, **E:** Sca-1, **F:** CD90.2, **G:** CD73, **H:** CD45, **I:** CD31, **J:** CD34, and **K:** c-kit.

We used flow cytometry to immunophenotype the cells (Fig 1A–1K), and found them to be positive for CD105 (endoglin, 97%, Fig 1C), CD29 (integrinβ1, Fig 1D), and Sca-1 (95%, Fig 1E). The cells expressed minimally other mesenchymal markers CD90.2 (3%, Fig 1F) and CD73 (2%, Fig 1G), and, other relevant markers CD45 (0%, Fig 1H), CD31 (0%, Fig 1I), CD34 (1%, Fig 1J), and c-kit (2%, Fig 1K). We previously documented the loss of c-kit expression during passage[2, 19].

### Differentiation stimuli decrease protein O-GlcNAcylation

**Fig 2:**
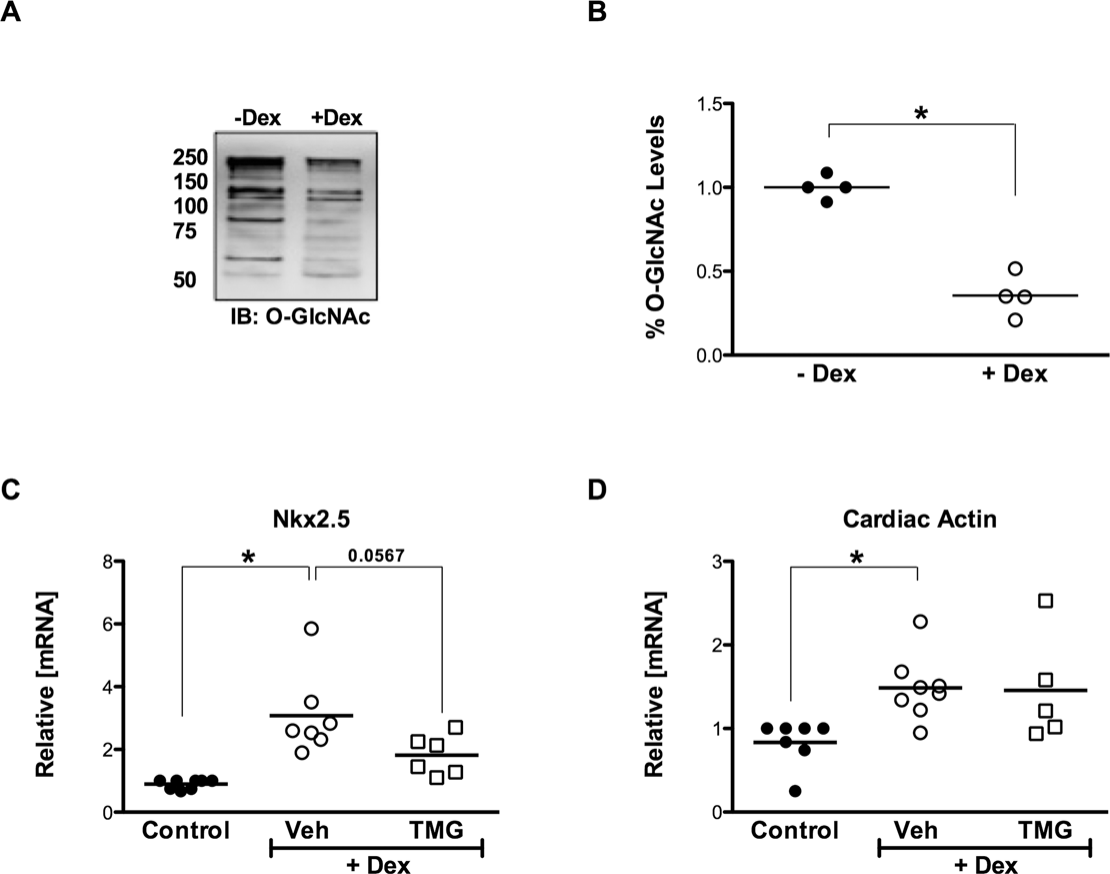
Dexamethasone-induced differentiation. **A:** Immunoblot showing decrease in total O-GlcNAcylated proteins 5 days post-differentiation (+Dex). **B:** Densitometry demonstrates a significant reduction in O-GlcNAc levels occur during dexamethasone-induced differentiation, relative to control, undifferentiated cells (-Dex). **C:** Differentiation-induced mRNA expression of early cardiac transcription factor Nkx2.5 is suppressed by maintenance of augmented O-GlcNAc levels during differentiation with the OGA inhibitor TMG. **D:** Early differentiation is also evidenced by enhanced cardiac actin expression, although unaffected by O-GlcNAcylation. Individual data points are plotted and the horizontal line represents the mean, n>/ = 4/group, *p < 0.05 vs–Dex/Control.

To study the initial response of murine cardiac mesenchymal cells to a differentiation stimulus, we exposed cells to dexamethasone (10^−9^ M) for 5 days, following which, total protein was harvested and immunoblotted for O-GlcNAc. Over time, differentiation decreased global O-GlcNAc levels (Fig 2A and 2B), which also resulted in a suppression of differentiation-induced Nkx2.5 upregulation (Fig 2C). Differentiating cells also had significantly increased cardiac actin transcripts (Fig 2D), though they were unaffected by increasing O-GlcNAcylation.

### There is an inverse relationship between O-GlcNAcylation and Nkx2.5 induction

**Fig 3:**
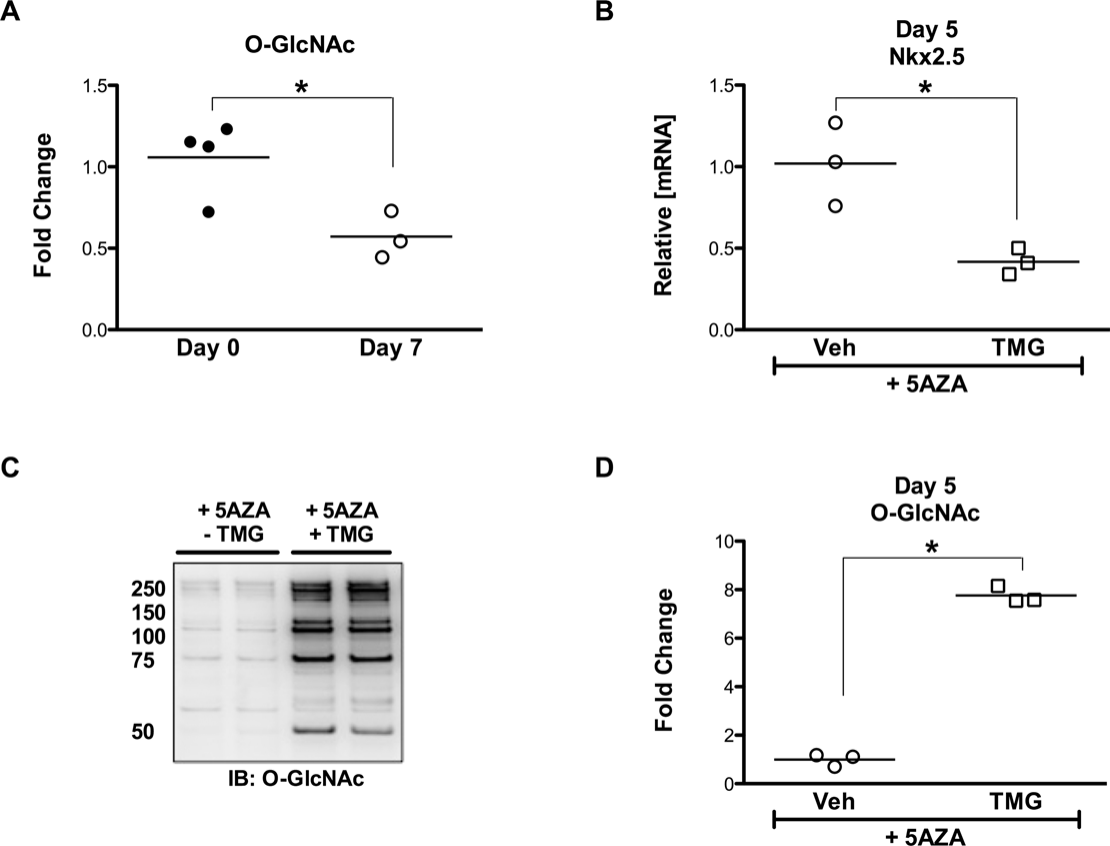
5-Azacytidine-induced differentiation. **A:** Densitometry showing significant reduction in O-GlcNAcylation at day 7 upon initiating differentiation stimuli in CSCs. **B:** Conversely, an enhanced O-GlcNAc status influences commitment to a cardiogenic lineage in terms of significantly reduced mRNA expression of an early cardiac transcription factor Nkx2.5. **C:** During this time, O-GlcNAc levels were maintained at an elevated degree as depicted in the immunoblot. **D:** Densitometric quantification showing significantly elevated O-GlcNAcylation due to TMG. Individual data points are plotted and the horizontal line represents the mean, n>/ = 3/group, *p < 0.05 vs day 0/-TMG.

**Fig 4:**
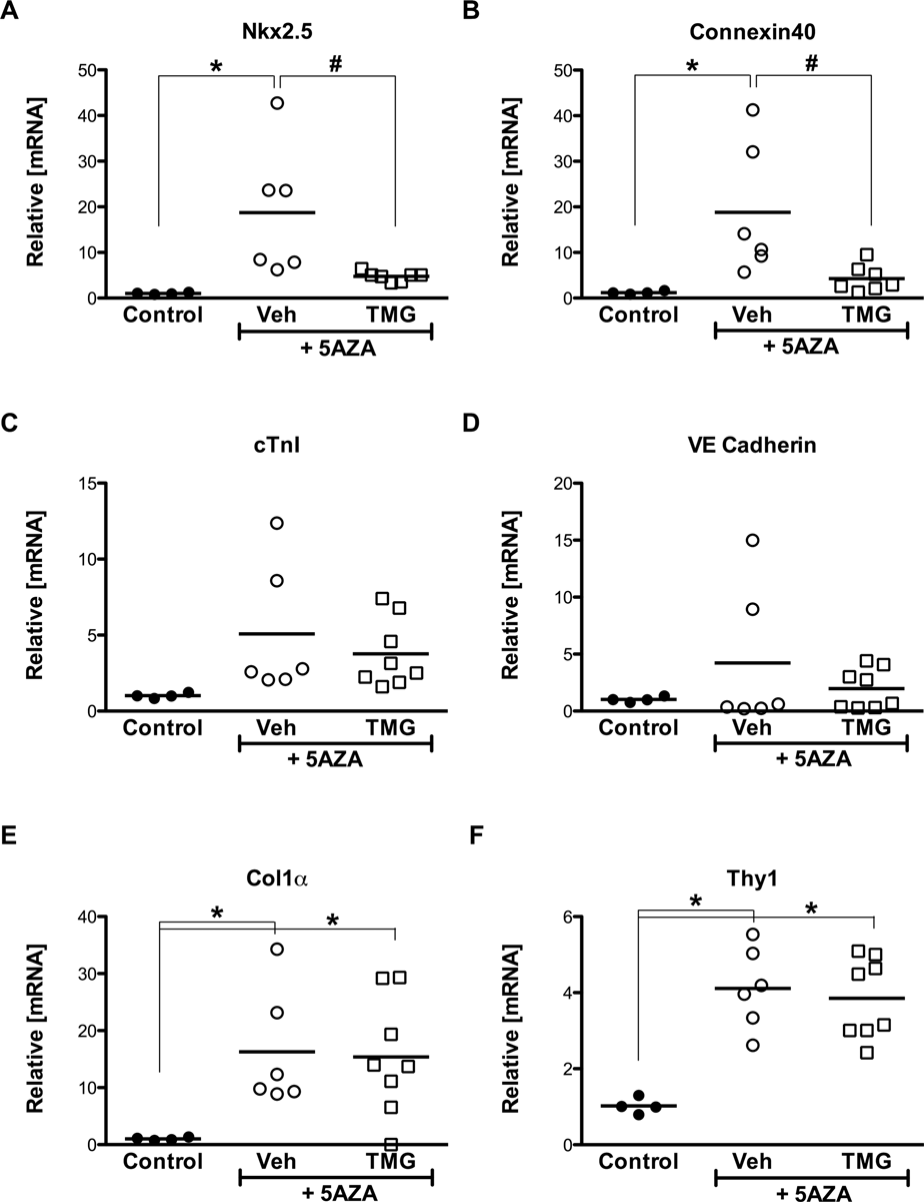
Enhanced O-GlcNAcylation limits cardiomyogenic commitment. **A:** Nkx2.5 expression following 5AZA-induced differentiation. **B:** Significant upregulation of connexin 40 during differentiation. **C:** Cardiac Troponin I, and **D:** Endothelial marker VE Cadherin did not change appreciably. **E, F:** Fibroblast markers Col1α and Thy1 are significantly induced during differentiation. Individual data points are plotted and the horizontal line represents the mean, n>/ = 4/group, *p < 0.05 vs Control; #: p < 0.05 vs Veh (+5AZA).

Protein O-GlcNAc levels diminished early (7 d; Fig 3A) and remained low over the period of the differentiation assay (21 d). A decline in Nkx2.5 manifested early (5 d; Fig 3B) coincident with augmented O-GlcNAc levels (TMG; Fig 3C and 3D). Significant induction of Nkx2.5 was evidenced upon exposure to 5-Azacytidine (21 d; Fig 4A); however, enhancing protein O-GlcNAcylation (TMG) suppressed this effect (21 d; Fig 4A). Moreover, the differentiation-induced expression of connexin 40 (Cx40), which is regulated by Nkx2.5, displayed a parallel reduction in response to elevated O-GlcNAc levels (Fig 4B).

The expression of cardiac troponin I (cTnI) and vascular endothelial cadherin (CD144) was found to be variable (Fig 4C and 4D). Interestingly, putative mesenchymal markers Col1α and Thy1 (CD90) were significantly expressed upon differentiation, but not unaffected in response to enhanced O-GlcNAcylation (Fig 4E and 4F).

### Protein O-GlcNAcylation reduces *Nkx2*.*5* promoter activation

**Fig 5:**
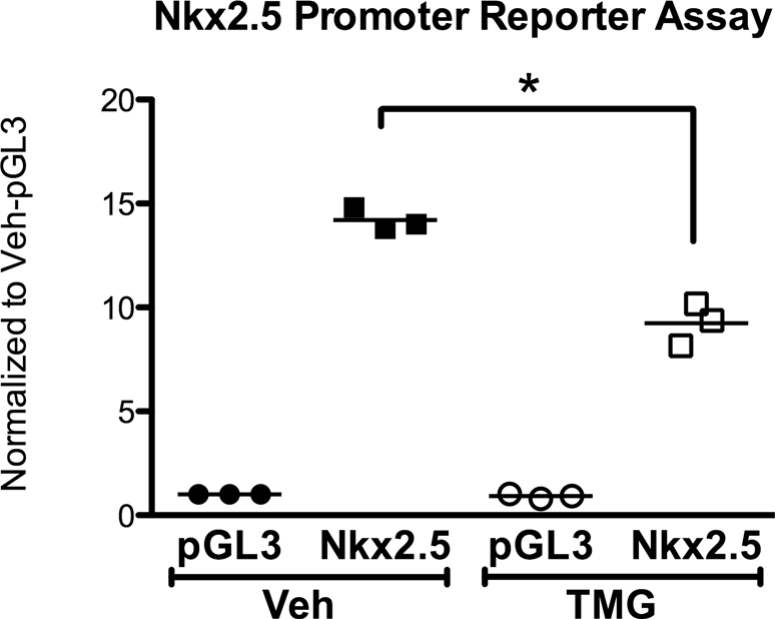
Protein O-GlcNAcylation inhibits *Nkx2*.*5* promoter activation. HEK cells treated with Veh or TMG (to enhance O-GlcNAc levels) were transfected with a plasmid containing the *Nkx2*.*5* promoter reporter. TMG significantly attenuated Nkx2.5 reporter activity. Individual data points are plotted and the horizontal line represents the mean, n = 3/group, *p < 0.05 vs Veh-Nkx2.5.

To interrogate a potential molecular mechanism for Nkx2.5 suppression by enhanced O-GlcNAcylation, we performed a promoter reporter assay after transfecting the *Nkx2*.*5* promoter reporter plasmid into HEK cells. Significant reduction in the *Nkx2*.*5* reporter activity was achieved in response to augmented O-GlcNAcylation (TMG; Fig 5). These results suggest that O- GlcNAcylation may impinge on the transcriptional regulation of the *Nkx2*.*5* promoter.

### O-GlcNAcylation potentiates smooth muscle actin (SMA) expression

**Fig 6:**
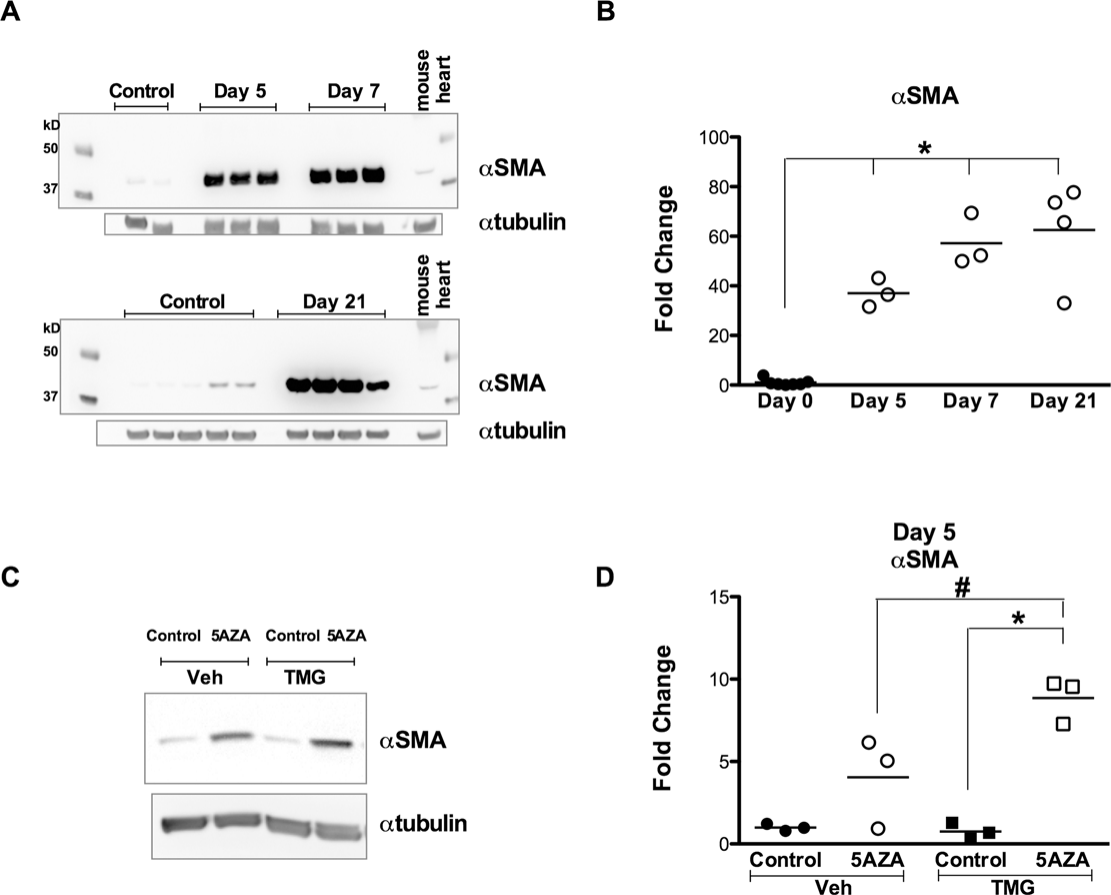
O-GlcNAcylation favors induction of smooth muscle actin. **A:** Immunoblot (with mouse heart lysate as positive control), and **B:** densitometric analysis demonstrating a robust early (day 5) and significant increase in smooth muscle actin (SMA) expression during differentiation. SMA remained elevated for extended periods of differentiation (7 to 21 d). **C:** Immunoblot, and **D:** densitometry revealing that higher O-GlcNAc levels (maintained with TMG) induced a significantly higher level of SMA expression 5 days post-differentiation than Veh treated differentiating cells. Individual data points are plotted and the horizontal line represents the mean, n>/ = 3/group, *p < 0.05 vs day 0/Control; #: p < 0.05 vs Veh +5AZA.

To further ascertain terminal differentiation, whole cell lysates were harvested at various time points for protein expression patterns. Neither VEGF2 nor cTnI were detected in differentiated or undifferentiated cells by immunoblotting, relative to whole mouse hearts (data not shown); however, smooth muscle actin (SMA) was found robustly upregulated by 5 days post-differentiation and persisted at later time points (7 d, 21 d; Fig 6A and 6B). Notably, enhancing protein O-GlcNAcylation (via TMG) resulted in a further upregulation of SMA relative to Veh treated differentiating cells (Fig 6C and 6D).

### Differentiation stimuli induce autophagy, which is not affected by O-GlcNAcylation

**Fig 7:**
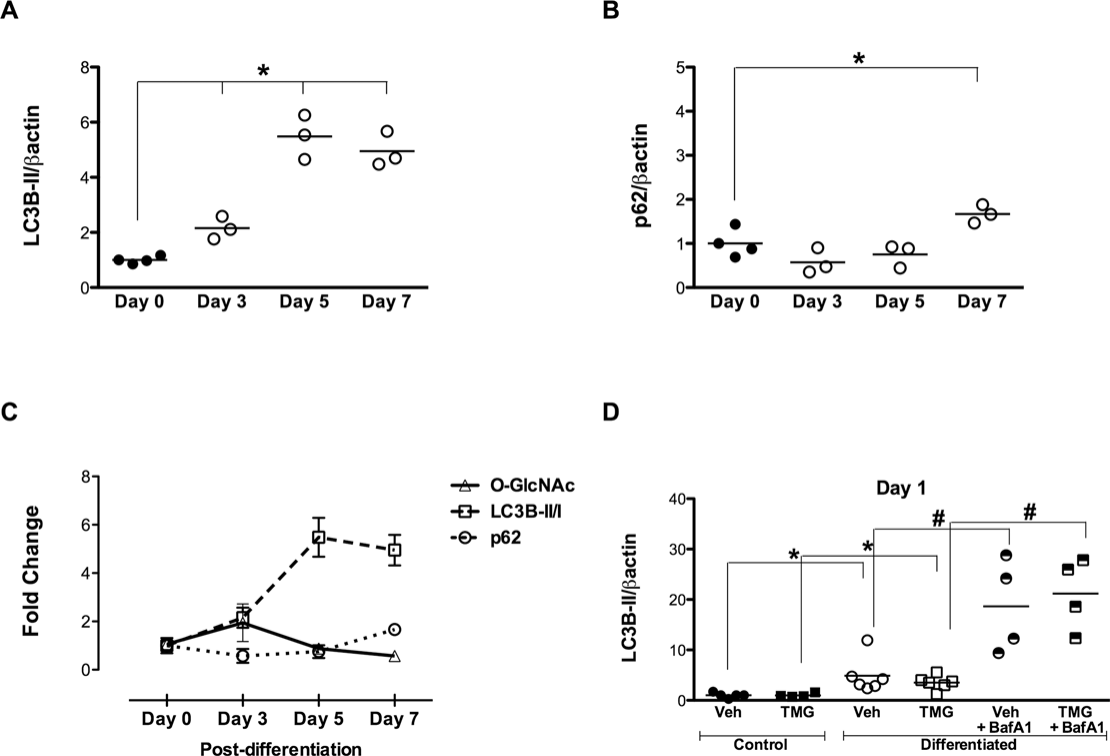
Enhanced O-GlcNAcylation does not affect differentiation-induced autophagy. Immunoblot and densitometric measurements examining **A:** significant induction of autophagosome-associated LC3B by day 3 of differentiation, occurring in parallel with **B:** a simultaneous decline in p62/SQSTM1 protein (by 44% on day 3) and significant stabilization by day 7. **C:** A temporal relation between O-GlcNAc levels in differentiating CSCs is displayed with components of autophagy signaling. **D:** Autophagic flux is intact during differentiation as evidenced by employing a flux inhibitor Baf A1 to verify autophagy activation in terms of accumulated LC3B-II. Individual data points are plotted and the horizontal line represents the mean, n = 3-4/group, *p < 0.05 vs day 0/respective Control; #p < 0.05 vs respective Differentiated.

During differentiation, autophagy may be required for morphological and structural changes necessary for cellular remodeling[20], and autophagy occurs in adult epidermal, dermal, and hematopoietic stem cells[21]. The involvement of autophagy in the differentiation of adult cardiac mesenchymal cells remains unknown. To examine whether autophagy was activated during differentiation, protein lysates from early differentiating cells were probed for lipidation of LC3B. By day 3, an initial and significant accumulation of autophagosome-associated LC3B-II had occurred (Fig 7A), concurrent with a ~44% reduction in p62/SQSTM1 abundance (Fig 7B), indicative of normal autophagic clearance. High levels of LC3B-II were also observed on days 5 and 7; p62/SQSTM1 returned toward baseline by day 5 and was significantly higher on day 7 (Fig 7A and 7B). To test further whether differentiation enhanced autophagic flux, Bafilomycin A1 was used during differentiation to inhibit the autophagosome-lysosome fusion step. Differentiating cells displayed higher levels of LC3B-II, which was increased further upon lysosomal blockade, revealing that autophagic flux is enhanced during differentiation, but independently of O-GlcNAcylation (Fig 7D). Thus, we also established that autophagy is activated in differentiating adult cardiac mesenchymal cells, reminiscent of the requirement of autophagy during embryonic development and cardiogenesis in heart progenitors[22]. Moreover, we provide evidence that increased O-GlcNAc levels do not interfere with autophagic flux in the context of differentiation.

### Enhanced O-GlcNAcylation does not affect proliferation or chemotaxis

**Fig 8:**
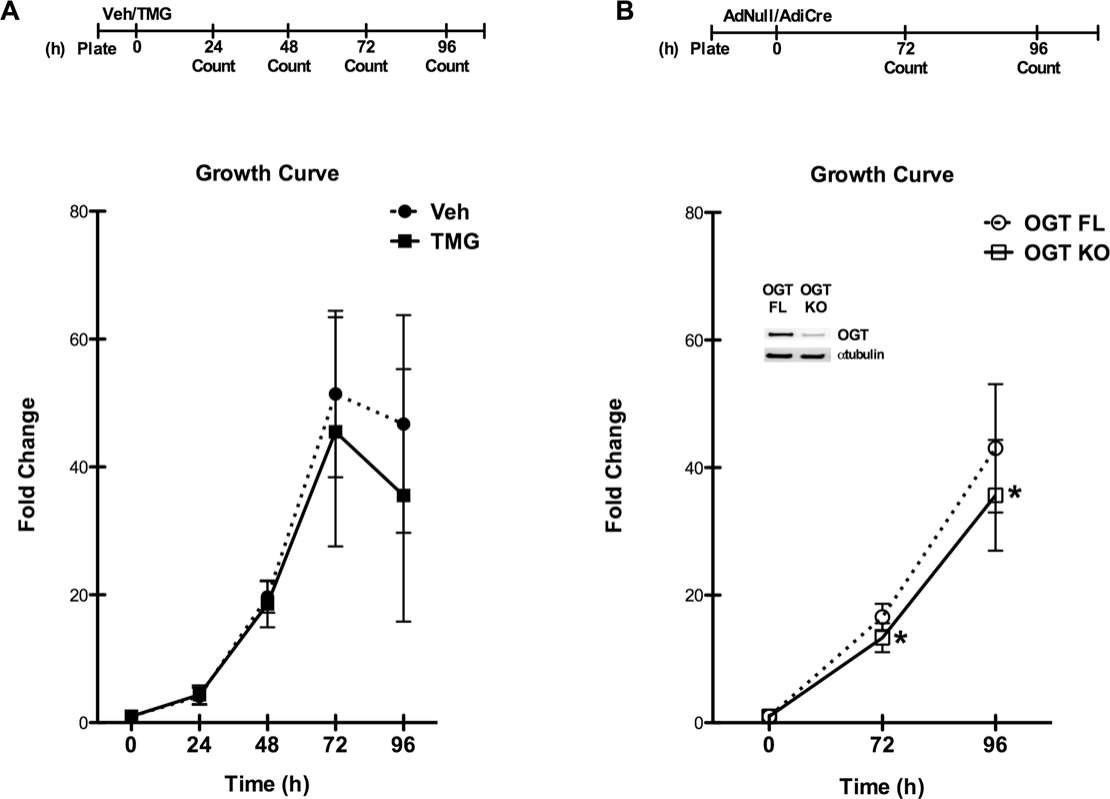
O-GlcNAc-primed CSCs maintain a normal growth profile, but loss of *Ogt* suppresses proliferation. **A:** Growth curves of CSCs maintained in culture with TMG demonstrate that augmented O-GlcNAc levels do not affect the characteristics of normal population growth. **B:** *Ogt* deletion results in significantly reduced cell proliferation. **Inset** indicates loss of OGT protein and diminished O-GlcNAc levels in the OGT KO cells. n = 3/group, *p < 0.05 vs OGT FL.

Given our previous publication showing that enhanced O-GlcNAcylation promotes cell survival, and our present results indicating that O-GlcNAcylation antagonizes commitment to a cardiomyogenic fate, it was important to determine whether other aspects of cell competence might also be affected. To this end, we treated cells with TMG or Veh and monitored cell proliferation (Fig 8A), and found no significant differences between the two groups as depicted by comparable cell numbers in the lag and exponential phases, with slight variability in the stationary phase. Next, we generated *Ogt* deficient cells to determine whether *Ogt* was required for cell proliferation, and found a mild, but statistically significant, impairment in proliferation (Fig 8B). Thus, *Ogt* performs a minimally permissive role in cell proliferation; however, increasing O-GlcNAcylation does not affect cell proliferation.

**Fig 9:**
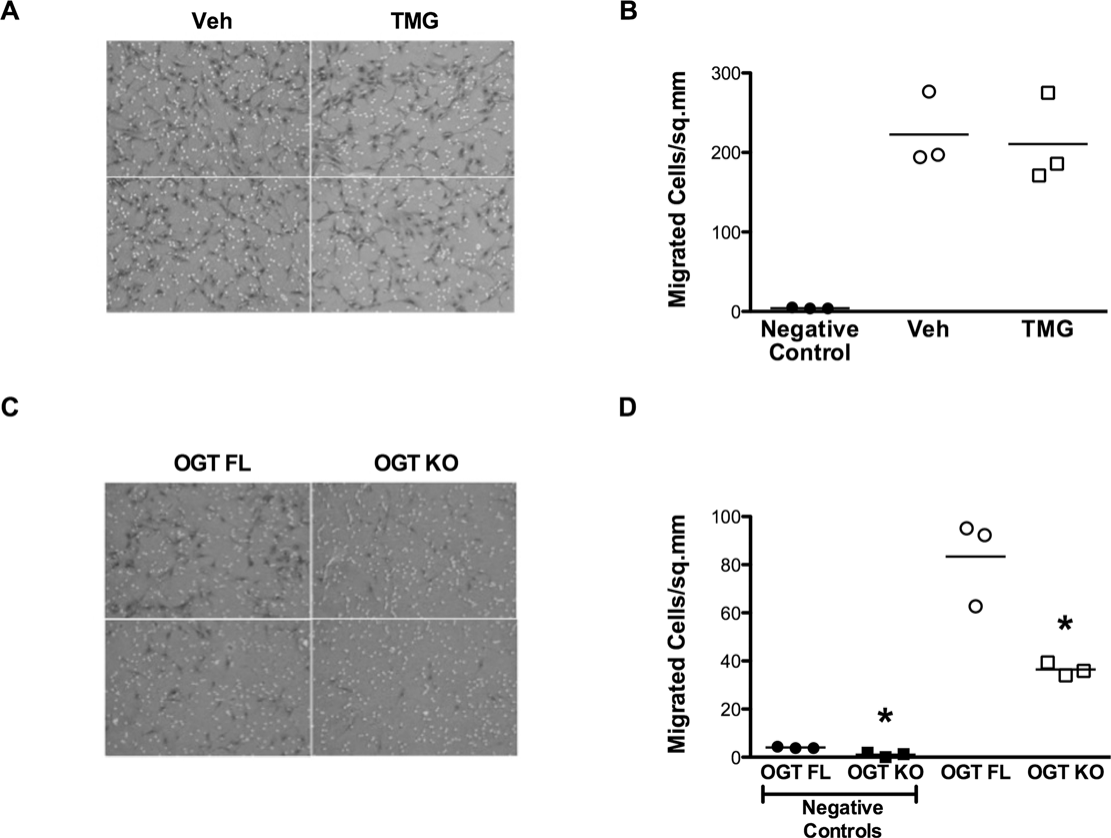
*Ogt* is at least partially permissive for proliferation and migration. **A:** Representative image (magnification 10x) showing cell staining for a transwell migration assay performed after enhancing O-GlcNAc levels, and **B:** Quantification of the migrated cells demonstrates that increasing protein O-GlcNAcylation does not alter cell motility relative to untreated cells. Negative controls are also shown. **C:** Cell staining is shown (magnification 10x) for cells carrying floxed *Ogt* and the effect of gene deletion in a migration assay, quantified in **D:** demonstrating that loss-of-function for *Ogt* significantly reduces the number of cells that can migrate. This effect is also borne out in negative controls. Individual data points are plotted and the horizontal line represents the mean, n = 3/group, *p < 0.05 vs Veh/OGT FL.

Cellular migration/chemotaxis is another important index of cell competence. We observed no significant migratory difference in cells with high O-GlcNAc levels (due to TMG) compared with Veh treated cells (Fig 9A and 9B). Conversely, half as many *Ogt* deficient cells migrated to the stimulus (Fig 9C and 9D). Unlike the modest differences observed with *Ogt* deletion in the proliferation experiments, *Ogt* apparently plays a more significant role in the ability of the cells to migrate to chemotactic stimuli.

## Discussion

Previous studies demonstrate that the level of protein O-GlcNAcylation changes as cells differentiate, suggesting O-GlcNAc levels may affect differentiation [23–28]. Yet, little is known about the influence of O-GlcNAcylation on cardiac lineage commitment. In primitive murine embryonic stem cell (ESC) cells, protein O-GlcNAcylation exerts negative regulation of self-renewal and pluripotency by modifying Oct4 (as well as Sox2), thereby influencing transcriptional activity of its downstream targets[23]. A decline in global O-GlcNAc levels occurs in mouse ESC-derived embryoid bodies (EBs), correlating with increased expression of cardiac-specific β-MHC protein[29]. Moreover, enhancing O-GlcNAc levels blocks cardiomyogenesis and was associated with lower numbers of cardiac ‘precursor’ cells. Augmenting protein O-GlcNAcylation in EBs inhibits the specification of cardiac and endothelial, but not hematopoietic, lineages[29]. In contrast, global changes in O-GlcNAc levels were not detected during the differentiation of human pluripotent stem cells (hPSCs). During spontaneous or directed differentiation of hPSCs, enhanced O-GlcNAcylation did not affect expression of markers for cardiac-related mesoderm (HAND1, Nkx2.5), visceral-related endoderm (GATA6, AFP), and trophectoderm (CDX2, BMP4)[30]; others found that induction of myogenesis in mouse C2C12 myoblasts was associated with reduced O-GlcNAcylation[24]. Thus, differentiation of various cell types is associated with unique roles of protein O-GlcNAcylation. The present study focused on whether O-GlcNAcylation affects cardiogenic commitment of cardiac mesenchymal cells.

Although protein O-GlcNAcylation may affect some differentiation programs[23–28], its influence on cardiogenesis has been described only recently by a single report[29]. In the present study, we investigated the differentiation program of adult cardiac mesenchymal cells–which are frequently used for autologous transplantation–and elucidated its relationship with O-GlcNAcylation. While the murine cells used here display a limited differentiation potential in that contractile cardiomyocytes fail to form, this feature is a reproducible phenomenon both *in vitro* and *in vivo*. Nevertheless, such partial differentiation (or commitment) is presumed to be an important component of the effectiveness of cell therapy. Here, we identified a differentiation profile involving enhanced Nkx2.5, SMA, Cx40, Col1α, and Thy1. Interestingly, antagonizing the reduction in O-GlcNAcylation during differentiation limited expression of cardiomyogenic, but not mesenchymal, markers. We also provided evidence for activation of autophagy during differentiation in adult cardiac mesenchymal cells, indicating that general cellular remodeling processes occur[22]; however, they were seemingly independent of O-GlcNAcylation in the present study.

Nkx2.5/Csx is a homeobox-containing cardiac transcription factor fundamental for cardiac development and differentiation; it is highly expressed in both embryonic and adult heart[31, 32], as well as having a minor and transient extracardiac expression[33]. Similar to our present results, a potential interaction between Nkx2.5 and O-GlcNAc was reported in mouse ESCs[29]. We investigated this phenomenon further at the molecular level by designing a promoter reporter assay to establish a link between protein O-GlcNAcylation and *Nkx2*.*5* activation. Indeed, higher O-GlcNAc levels suppressed *Nkx2*.*5* promoter reporter activation. We speculate that this may be indicative of putative transcriptional regulation of the *Nkx2*.*5* gene by O-GlcNAcylated transcription factors.

Our present data indicate that augmentation of O-GlcNAc levels can also suppress apparent induction of Cx40. Cx40 is a gap junction channel protein critical for cardiac electrical conduction, and is the most abundant connexin expressed in the conduction system[34]. Cx40 is thus expressed in atrial and conductive myocytes, but possibly also in interconnected fibroblasts in sinoatrial regions devoid of myocytes[35]. Although it is plausible that transcriptional activation of Cx40 could be a direct target of O-GlcNAcylation, another possibility exists: certain cardiac transcription factors, such as Nkx2.5, regulate the core promoter of the mouse *Cx40* gene[36]. Thus, it remains to be established whether O-GlcNAc regulates directly Cx40 expression or the effect is secondary to Nkx2.5.

*In vitro* cardiogenic differentiation studies typically do not assess mesenchymal (fibroblast-like) markers such as Col1α and Thy1; however, we detected a robust upregulation of both markers at multiple points following the differentiation stimulus. This, combined with robust expression of SMA, may indicate a propensity of at least a subpopulation of these cells to favor a myofibroblast-like state. Although enhancing O-GlcNAc levels did not affect Col1α and Thy1 expression, it reduced SMA mRNA expression; however, at earlier time points SMA protein was elevated.

Because we detected an early upregulation of differentiation markers in adult cardiac mesenchymal cells that was indicative of rapid cellular changes, we tested whether autophagy would be involved in driving this process. Autophagy is a homeostatic process for quality control of cellular macromolecules and organelles, but is also sensitive to environmental signals for cellular adaptation. It serves a conserved, critical regulatory role in development and differentiation (such as during erythropoiesis, lymphopoiesis and adipogenesis)[20], and is required for the maintenance of adult epidermal, dermal, and hematopoietic stem cells[21]. The concept of autophagy exerting control over the differentiation process has been described only recently in cardiac progenitors undergoing cardiogenesis[22]. Our present data indicate rapid induction of autophagosome-associated LC3B in differentiating adult cardiac mesenchymal cells, which appeared to involve enhanced autophagic flux. Yet, enhancing O-GlcNAcylation did not affect the engagement of autophagy. Thus, differences in global cellular remodeling are unlikely to explain the antagonistic effect of enhanced O-GlcNAcylation on cardiomyogenesis. It is plausible that both O-GlcNAcylation and autophagy regulate in parallel the commitment of cardiac mesenchymal cells.

In conclusion, protein O-GlcNAcylation is a regulator of adult cardiac mesenchymal cell function, capable of restricting cardiogenic differentiation potential. We predict that any potential functional benefit from O-GlcNAc-priming of cells for cell therapy would instead likely be derived from its potent prosurvival action[2]. While this may spare cells to instead serve as reservoirs for critical paracrine factors, a continual state of stress may not be conducive to allow these cells to commit to more specialized processes such as differentiation. Of course, it is becoming increasingly clear that these cells may not require significant differentiation to exert their beneficial effects.
